# Effects of Cognitively Based Compassion Training in the outskirts: A mixed study ^*^

**DOI:** 10.1590/1518-8345.5691.3531

**Published:** 2022-04-29

**Authors:** Flávia Cristiane Kolchraiber, Luiza Hiromi Tanaka, Lobsang Tenzin Negi, Ana Cristina Atanes, Káren Mendes Jorge de Souza

**Affiliations:** 1 Universidade Federal de São Paulo, Escola Paulista de Enfermagem, São Paulo, SP, Brasil.; 2 Bolsista da Coordenação de Aperfeiçoamento de Pessoal de Nível Superior (CAPES), Brasil.; 3 Emory University, Center for Contemplative Science and Compassion-Based Ethics, Atlanta, GA, Estados Unidos da América.; 4 Bangor University, School of Social Sciences, North Wales, PG, Inglaterra.

**Keywords:** Empathy, Meditation, Social Vulnerability, Complementary Therapies, Health Promotion, Community Participation, Compaixão, Meditação, Vulnerabilidade Social, Terapias Complementares, Promoção da Saúde, Participação Comunitária, Empatía, Meditación, Vulnerabilidad Social, Terapias Complementarias, Promoción de la Salud, Participación de la Comunidad

## Abstract

**Objective:**

to analyze the effects of Cognitively Based Compassion Training (CBCT®) among people in situations of social vulnerability.

**Method:**

a mixed, sequential and transformative study with the same QUAL→QUAN weight. Focus Groups were applied at the beginning (n=24) and three months (n=11) after CBCT®, to understand the participants’ knowledge about emotions, (self)care and stress situations. Content analysis was performed in the WebQDA software. The participants (n=65) were randomized into control (n=31) and intervention (n=34) to assess self-compassion, perceived stress, and positive and negative affects at three time moments. The mixed factorial ANOVA analysis considered within-participants (time) and between-participants (place and group) factors.

**Results:**

mean age (37), female gender (88%), single (51%) and black-skinned people (77%). The following thematic categories emerged before the course: “Reducing others’ suffering as a bridge to conscious self-care” and “Social vulnerability as a potentiator of low emotional literacy”. Subsequently, self-compassion and awareness of the mental states for social activism. The quantitative analysis showed a significant increase in self-compassion within-participants (p=0.003); group factor (p<0.001); perceived stress reduction (p=0.013); negative affects group factor (p=0.005); and increase in positive affects (p<0.001) within-participants.

**Conclusion:**

CBCT®️ exerted a positive effect on individual well-being and a positive impact on community engagement to promote social well-being in the outskirts. Brazilian Registry of Clinical Trials (RBR-3w744z.) in April 2019.

## Resumo

**Objetivo:**

analisar os efeitos do Treinamento Cognitivo de Compaixão (CBCT®) entre pessoas em situação de vulnerabilidade social.

**Método:**

estudo misto tipo transformativo sequencial com mesmo peso QUAL→QUAN. Grupos Focais aplicados no início (n=24) e três meses (n=11) após o CBCT®, para compreender o conhecimento dos participantes sobre emoções, (auto)cuidado e situações de estresse. A análise de conteúdo utilizou o *software WebQDA* ®. Os participantes (n=65) foram randomizados em controle (n=31) e intervenção (n=34), para avaliação de autocompaixão, estresse percebido e afetos positivos e negativos em três tempos. A ANOVA fatorial mista considerou fator dentre-participantes (tempo) e entre-participantes (local e grupo).

**Resultados:**

idade média (37), sexo feminino (88%), solteiras (51%) e pessoas negras (77%). Emergiram, antes do curso, as categorias temáticas: “Redução do sofrimento alheio como ponte para o autocuidado consciente” e “Vulnerabilidade social como potencializadora do baixo letramento emocional”. Em seguida, autocompaixão e consciência dos estados mentais para o ativismo social. A análise quantitativa demonstrou aumento significativo de autocompaixão dentre-participantes (p= 0,003); fator grupo (p< 0,001); redução do estresse percebido (p= 0,013); afetos negativos fator grupo (p= 0,005); e aumento dos afetos positivos (p< 001) dentre-participantes.

**Conclusão:**

o CBCT®️ teve efeito positivo sobre o bem-estar individual e promoveu impacto positivo no engajamento comunitário para a promoção do bem-estar social na quebrada. Registro Brasileiro de Ensaios Clínicos (RBR-3w744z.) em abril de 2019.

## Resumen

**Objetivo:**

analizar los efectos del Entrenamiento en Compasión Cognitiva (CBCT®) en personas en situación de vulnerabilidad social.

**Método:**

estudio mixto del tipo transformativo secuencial con el mismo peso QUAL→QUAN. Grupos Focales aplicados al inicio (n=24) y tres meses (n=11) después del CBCT®, para comprender el conocimiento que tienen los participantes sobre emociones, (auto)cuidado y situaciones de estrés. El análisis de contenido utilizó el *software WebQDA*. Los participantes (n=65) fueron aleatorizados en el grupo control (n=31) y experimental (n=34) para evaluar la autocompasión, el estrés percibido y los afectos positivos y negativos en tres momentos. El ANOVA factorial mixto consideró factor de participantes (tiempo) y entre participantes (lugar y grupo).

**Resultados:**

edad promedio (37), sexo femenino (88%), solteras (51%) y negras (77%). Las categorías temáticas que surgieron antes del curso fueron: “Reducción del sufrimiento de los demás como puente para el autocuidado consciente” y “La vulnerabilidad social como potenciadora de la baja alfabetización emocional”. Luego la autocompasión y la conciencia de los estados mentales para el activismo social. El análisis cuantitativo mostró un aumento significativo en la autocompasión de los participantes (p=0,003); factor grupo (p< 0,001); reducción del estrés percibido (p=0,013); afectos negativos factor grupo (p= 0,005); y aumento de los afectos positivos (p< 001) de los participantes.

**Conclusión:**

El CBCT®️ tuvo un efecto positivo en el bienestar individual y tuvo un impacto positivo en la participación de la comunidad para promover el bienestar social en la población de los barrios periféricos. Registro Brasileño de Ensayos Clínicos (RBR-3w744z.) en abril de 2019.

**Highlights:**

(1) Positive effect in a specific ethnic-cultural context and not studied before.

(2) Improvement in the participants’ individual well-being.

(3) Positive impact on community engagement to promote social well-being.

(4) Positive implication in the sociopolitical performance of volunteers considered activists

## Introduction

From the perspective of social determination, the existence of an intimate relationship between health and society is undeniable. Thus, all analyses of the health-disease-care process must necessarily consider the contradictions and social vulnerabilities that underlie the health problems and the way in which people with different social backgrounds access professional, popular, and informal care resources, including self-care^1^ ^-^ ^2^.

Social vulnerability is an interdisciplinary expression that refers to the sense of guaranteeing citizenship and to the fragility of social well-being, determined by a combination of sociopolitical and cultural factors that interfere with access to goods and resources to ensure the right to life with dignity^1^. Issues such as housing, income, usual sources of care, social networks, education, culture, individual and social expectations for the future are important social determinants of health. However, it is also necessary to attribute to the sphere of social (re)production the determination of the strains and strengthening instances of the health-disease-care process experienced by people, which can result in health problems^2^ ^-^ ^3^. 

With the understanding that care is inherent to human beings as a starting point, it can be admitted that social vulnerability reveals a complex process of (not) taking care (of oneself). In contexts of difficulties or lack of access to human rights, abandonment of care can represent humanity’s susceptibility to harms, distress or fatigue and finitude. Not taking care (of oneself) refers to the fragility and insecurity inherent to human beings, which can be manifested in the ontological, ethical, political, natural, cultural and social dimensions^4^ ^-^ ^5^. 

In order to expand the scope of interventions with an individual and specialized therapeutic approach, new models for promoting the well-being of populations with ethnocultural specificities are suggested^6^ ^-^ ^7^. In the field of Psychology, well-being comprises a complex and positive dimension of health that integrates cognition and affection. It is an ecological concept that comes from a broad system of intrinsic and extrinsic factors that influence the way or quality in which people lead their lives^8^ ^-^ ^9^.

Several studies have shown that well-being can act as a preventive factor against diseases. The presence of happiness and satisfaction with life implied a lower risk of mortality in healthy populations^9^ ^-^ ^13^. These results can be enhanced by training the mind in compassion^14^. Compassion is the sense of concern that arises when a person is faced with another’s suffering and the motivation to alleviate it. It is understanding the emotional state of other people and taking care of those who suffer. It also means promoting well-being in order to develop altruistic behaviors. When it is focused on the individual, it becomes self-compassion^15^ ^-^ ^18^. 

Recent studies indicate that training the brain in compassion through meditative practices results in changes in the body’s biochemical responses, such as a reduction in the levels of the inflammatory stress hormones and an increase in hormones linked to happiness^17^ ^,^ ^19^ ^-^ ^23^. This type of mind training expands behavioral domains, develops altruistic skills, and changes neural responses to suffering, which provides diverse evidence of neuroplasticity in the circuit underlying compassion and altruism^24^ ^-^ ^27^.

There are numerous compassion-based interventions. Programs employed as an effective emotion regulation strategy outside the traditional meditative context are associated with the endogenous production of positive affect, stimulating resilience to the suffering of others in the general population while promoting emotional connection and pro-sociality^19^ ^-^ ^21^ ^,^ ^24^ ^-^ ^28^.

Cognitively Based Compassion Training (CBCT®) is a secular program for training the mind in compassion with proven effectiveness in different groups; mostly, adult university students or specific groups in treatment of diseases. However, it is necessary to expand the proof of effectiveness with populations in social vulnerability situations. The peripheral communities of large urban centers, “the outskirts”, are part of this scope, and it is with them that we will develop this study^28^ ^-^ ^29^. The objective was to analyze the effects of Cognitively Based Compassion Training (CBCT®) among people in situations of social vulnerability.

It is understood that the current research brings with it theoretical-practical contributions due to its social relevance, innovation and advancement in scientific knowledge. Its contribution is also recognized so that people in situations of social vulnerability have access to practices that promote individual and collective well-being.

## Method

### Study design

This is a mixed-methods research study of the sequential transformative type and with the same weight between the QUAL→QUANT approaches. Data combination was performed through integration and occurred in the results and discussion. Choice of the transformative method is justified from the perspective of Paulo Freire’s epistemological theory, associated with the main researcher’s previous engagement in the communities participating in the study, in the direction of social justice and appreciation of the peripheral culture^30^ ^-^ ^33^.

The qualitative approach used was participant-action-research with the use of Focus Groups (FGs) and individual interviews (IIs) to understand the participants’ knowledge about feelings, emotions, compassion, stress and the CBCT® course. The following were applied: FGs before and three months after the intervention; and IIs immediately after the course. 

In the quantitative approach, a randomized and controlled study compared the benefits of the intervention on self-compassion, perceived stress, and positive and negative affects to the regular activities of Civil Society Organizations (CSOs). The dependent variables were the following: self-compassion, perceived stress, positive affects and negative affects. The independent variables were locus and group. A design with analysis between and among participants was used: 2 (Locus: CSOs vs. community) X 2 (Group: Intervention vs. Control) X 3 (Moment: T1 vs. T2 vs. T3). For this article, we used the results of all the aforementioned methods, except IIs in an integrated way. This approach focuses on the praxis, looking at and reflecting on the practice, considering ethics and well-grounded feelings^32^ ^-^ ^33^. 

### Study scenario

The study was carried out with assisted people and volunteers from two CSOs, in the region of Parelheiros, municipality of São Paulo, SP, Brazil. These are institutions that work on human rights and are located in peripheral areas. In partnership with five communities, they co-created the Center for Excellence in Early Childhood (*Centro de Excelência em Primeira Infância*, CEPI). During creation, difficulties were identified by the collective, such as: lack of focus, self-demand, and signs and symptoms of anxiety and stress for a more effective performance and contribution to well-being. During CEPI planning, the care for excellence was divided into dimensions and the contribution of meditative practices to the dimension of caring for the team was proposed to the researcher.

**Figure 1:**
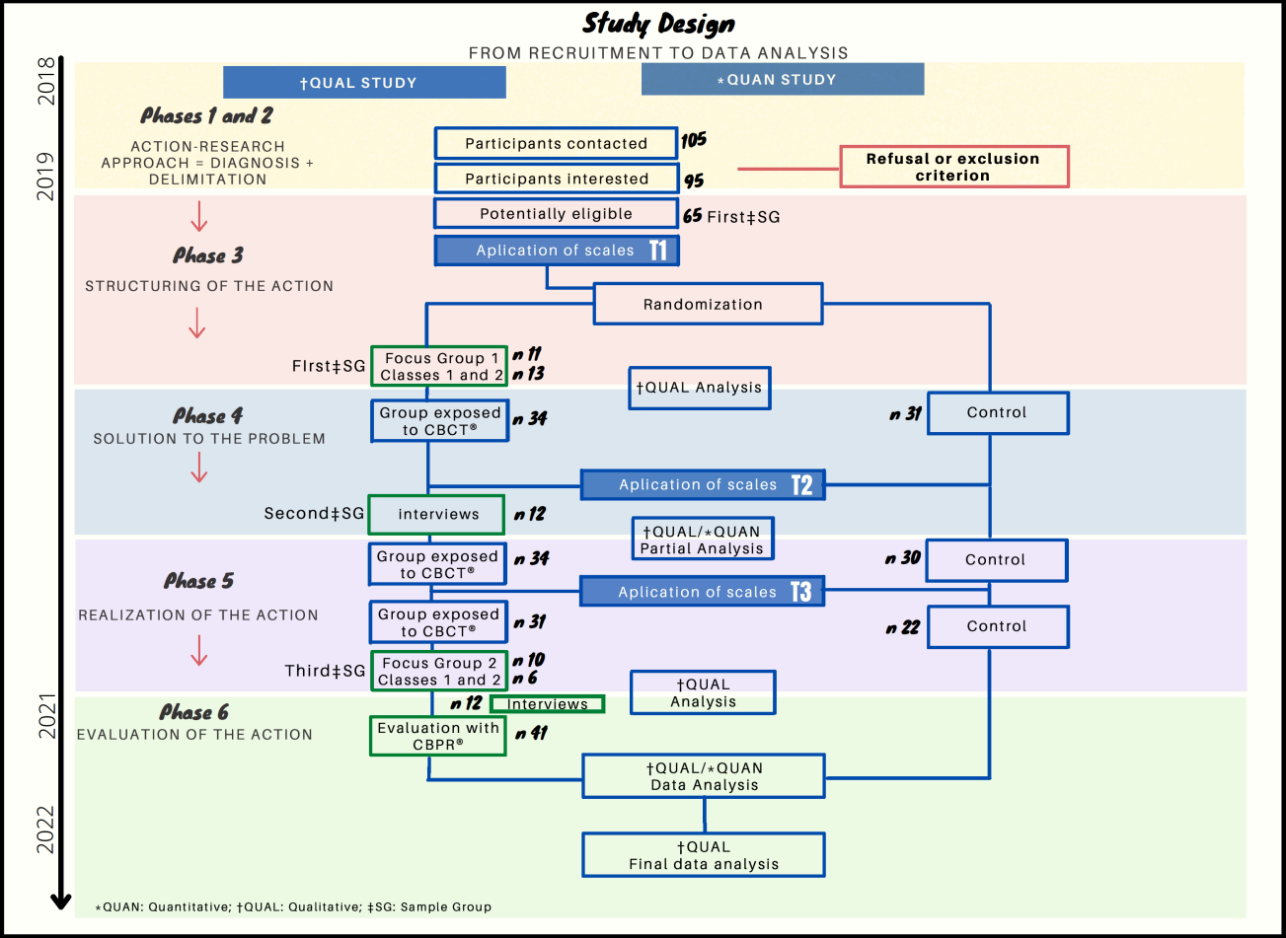
Flowchart corresponding to the inclusion, allocation, follow-up and analysis of the participants, adapted from CONSORT (n=65). São Paulo, SP, Brazil, 2018-2021

The study was approved by the Research Ethics Committee of the proposing institution in October 2018 under No. 2,906,902. It was conducted in accordance with the following recommendations: CONSORT (Consolidated Standards for Reporting Trials) for good clinical practices (Figure 1), COREQ (Consolidated criteria for reporting qualitative research) and Brazilian Registry of Clinical Trials (RBR-3w744z.) in April 2019.

### Participants, recruitment, randomization and sample size

The potentially eligible participants were recruited and randomized from May 2019 to June 2019 and contacted personally by the researcher or CSO volunteers. All information about the study was presented to the participants in the spaces of the CSO or in their homes. Eligibility criteria included: (1) age over 18 years old; (2) no previous practice in meditation; (3) residents of one of the five communities where the CSO operate; and (4) CSO volunteers. The exclusion criteria were people with severe mental illness undergoing treatment or with diagnosed cognitive impairments.

A total of 105 individuals were invited to participate in the research and 95 showed interest, most of them living outside the study area. 65 of them met the eligibility criteria, comprising the final convenience sample. After filling in the Informed Consent Form, the participants were randomly divided by the Randomizer software and distributed into an experimental group (CBCT® intervention) and a control group (CSO activity). 

### Materials

*Sociodemographic and clinical questionnaire*

Pre-tested with sociodemographic and economic variables (gender, age, ethnic-racial, marital status, schooling, number of children, income, number of people living on the income, time living in the region, religion, origin, employment contracts) and variables of interest (perception of mental and physical health and emotional trauma).

*Self-Compassion Scale* ^34^

A five-point Likert scale (from 1 = Almost never to 5 = Almost always) with 26 items assessing self-compassion about how people relate to themselves in difficult times. The items are divided into six subscales: self-kindness, severe self-criticism, sense of humanity, isolation, mindfulness, and over-identification. The overall score is calculated using the total of the subscales’ mean values after reversing the items from the negative subscales: 1, 2, 4, 6, 8, 11, 13, 16, 18, 20, 21, 24 and 25. The higher the score, the greater the self-compassion.

*Perceived Stress Scale* (PSS)^35^

A five-point Likert scale (from 0 = Never to 4 = Very often) with 10 items assessing the frequency of feelings and thoughts related to events and situations that occurred in the last month. Items 4,5,7 and 8 are inversely scored and added to all the items to calculate the total score, which varies from 0 to 40. A higher scores indicate more stress. 

*PANAS-X (Positive Affect Negative Affect Scale)-Adapted* ^36^

A five-point Likert scale (from 0 = Very little or not at all to 5 = Excessively) with 60 items assessing Positive Affects: basic positive affects, including joviality, self-confidence and attention; Negative Affects: basic negative affect, including fear, hostility, guilt and sadness; as well as other affective states, including shyness, fatigue, serenity and surprise. 

The scores for each dimension are calculated by adding up the ratings of all emotions included in each level and dividing the total by the number of emotions in each dimension, so that the scores vary from 1 to 5. In the sum, higher scores for Positive Affects indicate subjective well-being, disposition and enthusiasm and, for Negative Affects, they signal dissatisfaction with life, displeasure and subjective malaise.

### Data collection 

*Quantitative approach*

The participants’ sociodemographic-emotional profile was obtained using a self-administered questionnaire and scales in Google Forms® format. Individuals with difficulties reading and using computers were offered support. Two previously trained Nursing students conducted data collection with the researcher.

The Self-Compassion, PANAS-X and PSS scales were applied, all validated in Portuguese. Application took place in the initial phase (T1), immediately after the training (T2) and after 3 months (T3), as shown in Figure 1. 

*Qualitative approach*

All individuals were invited to participate in the initial FG (T1) and only those in the intervention group, for T3. Class 1 (n=11), Class 2 (n=13) at the pre-randomization phase, Class 1 (n=10) and Class 2 (n= 6), 3 months after the intervention. The FGs lasted two hours, were audio-recorded, transcribed (without the participants’ review) and conducted by a female interviewer, author of this paper, following a pre-prepared script. There was participation of two women observers, one of them being the main researcher. Both were nurses and experienced in this technique. The individual, video-recorded and transcribed interviews were carried out by an invited interviewer and immediately after the end of the intervention (T2) (n = 11). Both transcripts were validated by researchers, one of them being external to the team. The observers’ notes and the researcher’s observations described in the field notebook during and after data collection were added to the analysis. 

### Script for guiding questions

*T1 FG - Meeting to discuss the research object* 37. *How are the effects of stress on you? What is self-compassion and self-care?*

*T2 Interview - Conversation with a purpose* 38. *How was the experience of participating in CBCT®? What was most striking? Did anything change about you after the course?*

*T3 FG - How do you feel after the training in compassion? What effects did this program have on you and on the community?*

### Data analysis procedures

The final database was analyzed using the IBM SPSS® software, version 27.0. The data were analyzed by computing descriptive statistics (percentage, mean, standard deviation, asymmetry and kurtosis) and inferential statistics (mixed factorial ANOVA). For each variable of interest, mixed factorial ANOVA considered one within-participants factor (T1, T2 and T3) and two between-participants factors (locus and group).

**Figure 2:**
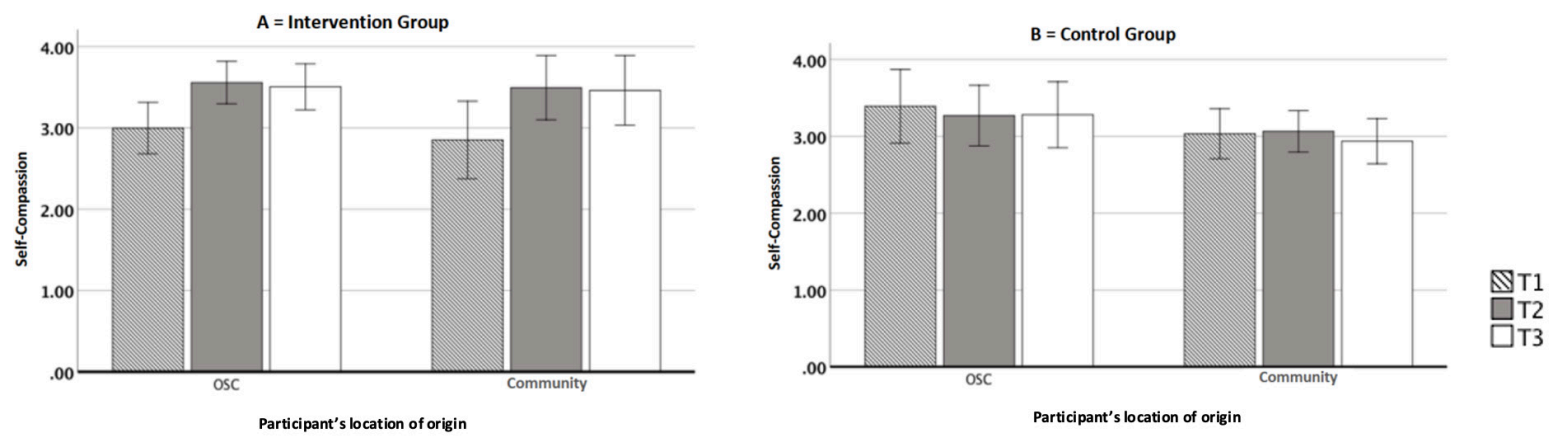
A) Differences in the self-compassion levels in the intervention group; B) Differences in the self-compassion levels in the control group of assisted people and volunteers from Civil Society Organizations, intervention group and control group (n=65). São Paulo, SP, Brazil, 2020

The qualitative material was organized in the *WebQDA*® software and submitted to categorical and thematic data analysis, performed in pairs: 1) Organization of the material: transcripts, observations, images, videos, notes, articles and quantitative database organized by study phase (Figure 1); 2) Categorization by sense nuclei; and 3) Interpretation: search for meaning, comparative analysis and data synthesis^39^.

### Intervention

CBCT® is a secular program of compassion meditation based on cognition and develops cultivation of compassion through meditative exercises^28^ ^,^ ^40^. It proposes to the participants the development of stability regarding attention and awareness of the nature of the mind (modules 1 and 2), progressing with specific and analytical compassion practices (modules 3 to 6).

The modules are as follows: (1) Attention to the present moment; (2) Insight into the nature of mental experience; (3) Cultivation of self-compassion and self-care; (4) Developing impartiality and equanimity; (5) Developing affection and gratitude; and (6) Understanding empathy and engaged compassion.

The meetings were held in the CSO space and lasted 2 hours, with weekly frequency and for nine meetings. They took place in a conversation circle format with projections, dynamics, texts for reading and videos for appropriation and dialog about the content and practices. 

The participants received a handout about the course and audios for practicing the meditations and were encouraged to write about experiences from the weekly practices. A social networking group was created for communication. All were invited to practice meditations outside the meeting hours for a mean of 15 minutes a day. The meetings were audio-recorded to ensure the content applied and fidelity to the CBCT® manual.

The instructor is certified by the Emory University Center for Contemplative Science and Compassion-Based Ethics, with long-standing experience in meditative practices and program teaching. 

The control group remained in the regular activities offered by the CSOs, physical activities, art workshops, reading, cooking, permaculture and conversation circles. At the end of the study, CBCT® was offered to the entire control group.

## Results

The results point out to QUAL→QUAN analyses in complementarity and revealed complex experiences, allowing other researchers to apply the model in different population groups. 

To meet the objectives of this study, the results were divided into two main parts. In the first one, the sample is characterized in relation to the demographic variables and to the perception of physical and mental health. In the second, inferential statistics are presented in order to test the effect of the intervention on the variables of interest, as well as further deepening, under the qualitative approach through three thematic categories based on 36 tree codes, derived from the data: (1) Reducing others’ suffering: a bridge to self-compassion and conscious self-care; (2) Social vulnerability as a potentiator of low emotional literacy; and (3) Self-compassion and awareness of mental states for social activism.

The curiosity to understand the different emotions, suffering itself and the desire to increase the repertoire to be able to help/take care of other people were the main motivations for the individuals to participate in the research. It is added that several participants reported the desire to overcome the fear of the unknown because they relate meditation to religious practice. 

### Characterization of the sample

In relation to the sociodemographic and economic data, the current study had the participation of 65 people (n_control_= 31, n_intervention_= 34). Their mean age was 37 years old (SD = 14.96), most of them were female (88%), single (51%) and black-skinned (brown and black) (77%), as shown in Table 1.

**Table 1:** Sociodemographic and economic data of assisted people and volunteers from Civil Society Organizations (n=65). São Paulo, SP, Brazil, 2020

Variable	Category	n	Percentage/Frequency
Gender	Male	8	12%
	Female	48	88%
Locus	CSO	24	52%
	Community	32	48%
Ethnicity	White	10	20%
	Brown	26	43%
	Black	18	34%
	Asian	2	3%
	Indigenous	0	0
Marital status	Stable union	10	17%
	Single	32	51%
	Married	12	25%
	Divorced	1	6%
	Widowed	1	1%
Children	0	17	29.2%
	1	17	23.1%
	2	6	15.4%
	3	8	12.3%
	4	2	6.2%
	5 or more	9	14%
Schooling	Complete HS	20	38%
	Incomplete HS	4	8%
	Complete ES	4	6%
	Incomplete ES	9	14%
	Never attended school	3	5%
	Complete HE	6	11%
	Incomplete HE	10	18%
Job	PF temporary contract	3	4%
	Entrepreneur/Legal Entity	7	11%
	I don’t have	27	45%
	Formal contract	10	20%
	No formal contract	9	20%
Family income	Less than 1 minimum wage	6	12%
	1 minimum wage	22	38.5%
	2 minimum wages	15	28%
	3 minimum wages	4	6%
	More than 3 minimum wages	9	1.5%
	No income	9	14%

*Current minimum wage = R$ 1,045.00, Brazil, 2020

Regarding the perception of the sample in relation to the physical and emotional aspects, 71% of the participants considered themselves slightly or moderately healthy, while 29% perceived themselves as healthy or very healthy. In relation to the emotional aspects, the majority (65%) indicated not having had any trauma, and 26%, 6% and 3% reported having had one, two and three traumas, respectively. 

As shown in Table 2, descriptive statistics were calculated for the variables of interest (self-compassion, perceived stress, negative affects and positive affects) for the three moments (T1, T2 and T3) and for the subgroups (CSO *vs*. community and intervention *vs*. control).

**Table 2:** Descriptive statistics of the self-compassion, perceived stress, negative affects and positive affects variables of assisted people and volunteers from Civil Society Organizations (n=65). São Paulo, SP, Brazil, 2020

Variable	Subgroup	M^||^	SD^¶^	Shapiro-Wilk sig.	Asymmetry (EP)	Kurtosis (EP)
*SC T1	CSO	3.08	0.70	0.65	-0.28 (0.44)	-0.11 (0.86)
	Community	2.90	0.59	0.74	-0.24 (0.46)	-0.52 (0.89)
	Intervention	2.91	0.71	0.74	-0.10 (0.42)	-0.52 (0.82)
	Control	3.10	0.56	0.69	-0.06 (0.48)	0.06 (0.94)
*SC T2	CSO	3.51	0.55	0.57	-0.04 (0.44)	-0.57 (0.86)
	Community	3.15	0.49	0.39	-0.41 (0.46)	0.37 (0.89)
	Intervention	3.52	0.51	0.89	0.12 (0.42)	-0.51 (0.82)
	Control	3.09	0.50	0.87	-0.37 (0.48)	-0.23 (0.94)
*SC T3	CSO	3.43	0.53	0.25	-0.01 (0.44)	-0.79 (0.86)
	Community	3.10	0.60	0.94	-0.13 (0.46)	-0.20 (0.89)
	Intervention	3.46	0.54	0.21	0.04 (0.42)	-0.80 (0.82)
	Control	3.02	0.56	0.47	-0.34 (0.48)	-0.56 (0.94)
PSS T1^†^	CSO	21.93	7.21	0.65	0.21 (0.44)	-0.65 (0.86)
	Community	23.50	6.63	0.66	0.33 (0.46)	-0.48 (0.89)
	Intervention	23.48	7.06	0.54	-0.04 (0.42)	-0.83 (0.82)
	Control	21.61	6.71	0.65	0.61 (0.48)	0.40 (0.94)
PSS T2^†^	CSO	18.93	1.08	0.37	0.44 (0.44)	-0.54 (0.86)
	Community	19.62	4.55	0.53	-0.17 (0.46)	-0.07 (0.89)
	Intervention	18.97	5.34	0.18	0.52 (0.42)	-0.08 (0.82)
	Control	19.65	4.98	0.65	-0.31 (0.48)	-0.62 (0.94)
PSS T3^†^	CSO	20.39	6.26	0.39	0.47 (0.44)	-0.08 (0.86)
	Community	21.46	1.09	0.90	-0.19 (0.46)	0.34 (0.89)
	Intervention	19.84	6.27	0.46	0.41 (0.42)	0.23 (0.82)
	Control	22.35	5.16	0.82	0.06 (0.48)	-0.44 (0.94)
NA T1^‡^	CSO	2.48	0.79	0.51	0.21 (0.44)	-0.74 (0.86)
	Community	2.63	0.84	0.46	0.17 (0.46)	-0.67 (0.89)
	Intervention	2.74	0.84	0.33	-0.10 (-.42)	-0.77 (0.82)
	Control	2.31	0.71	0.34	0.54 (0.48)	0.05 (0.94)
NA T2^‡^	CSO	2.11	0.70	0.002	0.87 (0.44)	-0.53 (0.86)
	Community	2.10	0.66	0.22	0.81 (0.46)	1.00 (0.89)
	Intervention	2.01	0.71	0.0004	1.26 (0.42)	0.67 (0.82)
	Control	2.22	0.61	0.84	0.25 (0.48)	0.02 (0.94)
NA T3^‡^	CSO	2.43	0.67	0.68	-0.003 (0.44)	-0.92 (0.86)
	Community	2.32	0.90	0.30	0.63 (0.46)	0.23 (0.89)
	Intervention	2.45	0.85	0.47	0.35 (0.42)	-0.26 (0.82)
	Control	2.28	0.64	0.63	0.05 (0.48)	-0.77 (0.94)
PA T1^§^	CSO	3.15	0.80	0.12	-0.59 (0.44)	0.26 (0.86)
	Community	3.00	0.76	0.01	-0.83 (0.46)	-0.40 (0.89)
	Intervention	3.14	0.85	0.01	-0.65 (0.42)	-0.22 (0.82)
	Control	3.00	0.68	0.07	-0.90 (0.48)	0.36 (0.94)
PA T2^§^	CSO	2.74	0.56	0.24	-0.02 (0.44)	-0.69 (0.86)
	Community	2.64	0.64	0.68	-0.31 (0.46)	-0.01 (0.89)
	Intervention	2.85	0.61	0.45	-0.15 (0.42)	-0.78 (0.82)
	Control	2.49	0.53	0.07	-0.81 (0.48)	-0.02 (0.94)
PA T3^§^	CSO	3.44	0.69	0.51	0.00 (0.44)	-0.03 (0.86)
	Community	3.18	0.76	0.26	-0.61 (0.46)	0.41 (0.89)
	Intervention	3.46	0.79	0.32	-0.55 (0.42)	0.54 (0.82)
	Control	3.11	0.59	0.14	-0.76 (0.48)	0.09 (0.94)

*Self-Compassion; ^†^Perceived Stress Scale; ^‡^Negative Affects; ^§^Positive Affects; ^||^Mean; ^¶^Standard Deviation

### Effects of the intervention according to quantitative and qualitative approaches

A series of mixed ANOVA analyses was performed in order to verify the effect of the intervention on the variables of interest, as well as the differences between the groups. The results of these analyses are presented below, taking into account the assumptions for this parametric analysis.

### Self-compassion

The self-compassion variable presented normal distribution at the three levels (T1, T2 and T3). Presence of only one outlier at T2 was observed in the community group, choosing to keep it in the analysis considering that ANOVA is robust in relation to small violations. The homogeneity assumption was complied with, but homoscedasticity was violated. Therefore, for the within-subject effects, the Greenhouse-Geisser statistic was reported.

A significant difference was observed for the within-subjects factor: [F(1.674, 83.701) = 7.123, p = 0.003, ηp2 = 0.13], as well as a significant interaction with the group factor: [F(1.829, 83.701) = 10.61, p < 0.001, ηp2 = 0.18]. Seeking to better understand the interaction between self-compassion and the group, simple comparison tests were performed. For the intervention group, a significant effect on self-compassion was observed: [F(1.486, 44.582) = 17.35, p < 0.001, ηp2 = 0.37]. A significant increase in self-compassion was observed between T1 and T2 (p < 0.001), and between T1 and T3 (p = .001), as shown in Figure 2. 

95%CI error bars

### Reducing others’ suffering: a bridge to self-compassion and conscious self-care

This category was based on the perception of self-compassion and self-care. For the participants, this took place in a consonant way, for helping, caring for and being with people. The recognition of ancestral consciousness and the success story itself resonates with most of the people in the midst of hostile realities, political consciousness and the social situation as determinants for the search for self-care. A number of self-care practices were identified, such as: friends, literary reading, reflection on everyday experiences, spirituality, contact with nature, creation, breathing and relaxation practices, belonging to a collective, psychotherapies, verbal expression and, finally, feeling/allowing oneself to be taken care of and to dialog.

*I believe that we are in a process of building the human being. Human beings were not born ready, they were not born perfect. So, when we refuse to listen to the other, we are not denying the other’s right to speak, we are also denying the process of listening and building as a human being.* (Tokyo)

*I am who I am, I’m the one who will carry myself forever, no matter that I have a husband, mother, children, and regardless of these relationships. You have to be okay, even for you to be okay with other people.* (Pega-pega)

### Perceived stress

The perceived stress variable showed normal distribution in the three levels (T1, T2 and T3). Presence of only one outlier at T2 was observed in the intervention group and t-Ωhree outliers at T3 in the CSO and intervention groups, choosing to keep them in the analysis considering that ANOVA is a robust analysis for small violations. Compliance with the homogeneity assumption was observed; however, homoscedasticity was violated. Therefore, for the within-subject effects, the Greenhouse-Geisser statistic was reported. Mixed ANOVA 2 (Locus: CSO vs. community) X 2 (Group: intervention vs. control) X 3 (Perceived stress: T1 vs. T2 vs. T3) only indicated a significant difference in the within-subjects factor: [F(1.586, 91.959) = 5.14, p = 0.013, ηp2 = 0.08]; with a reduction at T1 and T2 (p = 0.003), T1 and T3 (p = 0.03).

### Social vulnerability as a potentiator of low emotional literacy

This category was based on the question about how the participants perceive feelings, emotions and sensations in relation to stress. The perception of conflicting emotions was mentioned, primarily expressed by the social condition experienced due to not breaking the vicious cycle in the family history, unresolved relationships, household routine, living in a stressful environment, perpetuation of violence in its different forms, excess of activities with expectation of change in the conditions, in precarious public transportation and the time spent on it, disrespect and social injustice (racism, gender relations, poverty, inequalities, being peripheral, being seen as stressed or worthless). 

In a second place, it was expressed by the issue of not knowing how to name feelings, emotions and sensations; a condition recognized by excess of thoughts, mental distress, anxiety, stress, reactivity, silencing, isolation, nervousness, aggressiveness, self-demand, irritability for being contradicted and not feeling listened to, body aches, lack of sleep and excessive dedication to caring for the other.

*I’m a person who considers myself stressed 24/7. Due to the family issue, to the family environment, to my everyday routine. Not because of the people, but the routine I have of waking up early, arriving late, traveling around São Paulo to study.* (Araçuaí) 

*Not knowing how to name the things I feel, including stress, for not having this perception of the sensitive field, which is very abstract, very subjective. And it’s something that’s always on the other. You have to know how to qualify in the other. The person is moody today, the person is stressed. Understood?! They did such a thing, they are this. But when it’s on us, we don’t name it.* (Parati)

### Negative affects

The variable presented normal distribution at the three levels (T1, T2 and T3), there was a violation of normality at T2 in the CSO and intervention groups. However, these were not significant violations, opting for not transforming the variable. Presence of only five outliers was observed at T2, which were maintained in the analysis, as their removal did not imply changes in the results. Compliance with the homogeneity and homoscedasticity assumptions was observed. A mixed ANOVA 2 analysis was performed (Locus: CSO vs. community) X 2 (Group: intervention vs. control) X 3 (Negative Affects: T1 vs. T2 vs. T3). 

**Figure 3:**
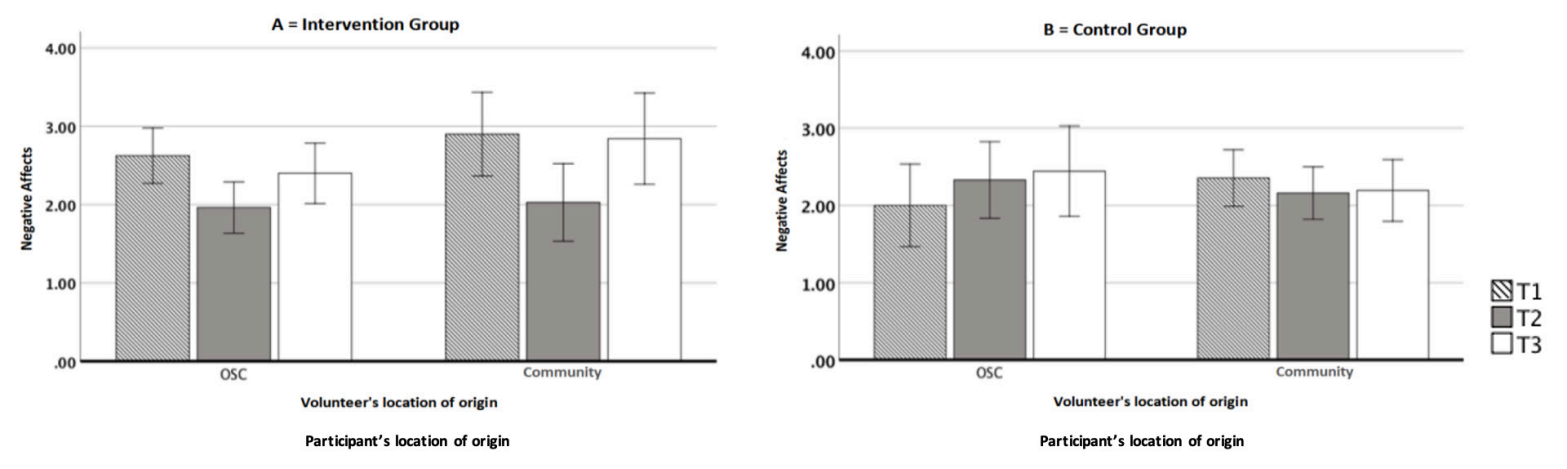
A) Differences in the levels of negative affects in the intervention group; B) Differences in the levels of negative affects in the control group of assisted people and volunteers from Civil Society Organizations in the intervention group and the control group (n=65). São Paulo, SP, Brazil, 2020

There was a significant difference in the within-subjects factor: [F(2, 1000) = 5.16, p = 0.007, ηp2 = 0.09]; The interaction between negative affects and group was significant: [F(2, 1000) = 5.61, p = 0.005, ηp2 = 0.10]; Seeking to better understand the interaction between negative affects and group, tests of simple comparisons were performed. For the intervention group, a significant effect on negative affects was observed: [F(2, 60) = 10.43, p < 0.001, ηp2 = 0.26]. There was a significant reduction in negative affect between T1 and T2 (p < 0.001) and a significant increase between T2 and T3 (p = 0.019), as shown in Figure 3.

95%CI error bars

### Positive affects

This variable presented a normal distribution violation at the T1 level for the community, intervention and control subgroups; and at the T2 level for the control group. Presence of eight outliers was observed between T2 and T3, which were removed. After its removal, the variable presented normal distribution across the three levels. Compliance with the homogeneity and homoscedasticity assumptions was observed. A mixed ANOVA 2 analysis was performed (Locus: NGO *vs.* community) X 2 (Group: intervention *vs*. control) X 3 (Positive Affects: T1 *vs*. T2 *vs*. T3). 

A significant effect of the group factor was observed: [F(1, 51) = 7.66, p = 0.008, ηp2 = 0.16], with the intervention group presenting the highest mean. There was also a significant difference in the within-subjects factor: [F(2, 82) = 33.07, p < 0.001, ηp2 = 0.45]. There was a significant reduction between T1 and T2 (p = 0.001) and a significant increase between T2 and T3 (p < 001). 

### Self-compassion and awareness of mental states for social activism

This category was based on the effects of CBCT® on the participants and their communities. The program was unanimously assessed positively by the recognition of the modules in everyday life (cited in the reports below) and by the deconstruction of preconceptions. The need for the course as a recurring training was pointed out due to its benefits added to a dynamic methodology, clear material, accessible practice and kind instructor.

CBCT® provided a space for dialog to bring up common issues, establish reflections on what is individual and collective, and deepen on the feeling/being. In addition to that, it contributed to stress and anxiety reductions and became a space for the production of critical-reflective knowledge. Emotional regulation was evidenced by the importance of living the present moment, noticing the functioning of the mind and the reduction of judgments and self-acceptance. Valuing who is close and the desire to expand learning for their own benefit and within the community were presented as impacts on social transformation. In this category, self-compassion and self-care senses were revealed towards the development of empathy and engaged compassion: 

*You living more and more in the present even though you know that today I’m like this, tomorrow I’ll be different, yesterday I was someone else. This marked me a lot in the meditation.* (Pega-pega)

*When we start to see our thoughts that are a little messy, then the way we focus all of them changes and we learn to know how to talk with what we’re bringing to mind. So it also helped in this part, that’s it, bringing more focus, seeing myself in the midst of the reality I live in.* (Lego)

*An example that I also took and took down note of was that we always depend on each other, we can’t only want to have compassion for ourselves too, we need to have compassion for others, understand them.* (Bahia)

## Discussion 

The hypothesis of this study was that the CBCT® applied in the “outskirts” improves self-compassion, perceived stress, the perception of feelings and emotions, and the individual and social well-being of CSO assisted people and volunteers. 

As the first study to investigate the effects of the program on this population, our results showed that CBCT® was effective regardless of the participant’s location, community (assisted people) or volunteering, with significant correlations being observed that will be discussed in the light of the theoretical model on studies about compassion and Freire’s transformative education.

The sample under study, based on an intersectional approach, evidences the predominance of females, black (brown or black) self-reported ethnicity, unemployed, with children, and income of one minimum wage, which are sociodemographic data characteristic of the peripheral population of São Paulo-SP, Brazil^41^ ^-^ ^42^. The majority reported their perception of physical and mental health as little or moderately healthy and absence of traumas. According to the trauma resilience model, people’s perception of cumulative trauma (colonialism, racism, homophobia, etc.) is low, possibly because it is something that happens daily for a long period of time^42^ ^-^ ^43^.

Self-compassion increased over time for the intervention participants. This significant increase from the beginning to the final CBCT® measurement remained the same after three months of the course. It was not the case for the control group, which maintained the same level at all times. From the point of view of self-compassion, the course exerted a positive impact on the participants. It brought to light the perception of their own distress and ways to alleviate it, one of them being reducing others’ suffering as a bridge to self-compassion and conscious self-care. 

A number of studies show that self-compassion is positively related to social ties. The compassion we direct to ourselves is the one we offer to other people^18^ ^,^ ^44^. The connection with the other’s suffering drives the group to a loving dialog with the other or with oneself and allows practical reflections for more resilient states^18^. The greater the self-compassion, the greater the compassion for the others and the greater the well-being and social transformation^45^ ^-^ ^46^.

In relation to the effect on the perception of situations as stressful, there was a significant reduction over time in both groups, with a greater reduction between the initial and final moments of the course in the intervention group. The similar perception for everybody suggests that mental distress is common to all beings^45^ ^-^ ^46^. The connection between people who are part of the same community and share living spaces can contribute to the reduction of social stress^47^. Another interpretation is that social vulnerability sustains the effects of stress for a longer period of time, as well as physical and psychological symptoms^48^.

A number of studies show that the perception of stress is not necessarily something negative, but a perception for self-regulation. It is worth remembering that the PSS scale asks the reader for an assessment of the last month, which contributes to the understanding that social vulnerability can enhance low emotional literacy. It is necessary to understand both the nature of the social situation and the means to transform realities, which takes time^49^.

Negative affect had a significant reduction over time for the CBCT® group from the initial to the final phase of the course, although with an increase between the end of the course and the measurement after three months. Recognition of the modules in everyday life appears in the participants’ reports, suggesting a challenging path to self-compassion and individual transformation. Structured inequalities, such as racism, misogyny and homophobia, are like heart diseases that take longer to be discovered and/or treated^49^ ^-^ ^51^. This requires identifying the suffering itself, its perception and an approach to unpleasant experiences^52^; with compassionate acceptance to recognize suffering in its raw state, relating to it in a more conscious way and allowing the support of conscious and non-judgmental attention^27^ ^,^ ^50^.

A significant increase in positive affect was observed for the intervention group, regardless of whether the participants belonged to the category of community or volunteer people. In relation to time, there was a reduction from the beginning to the end of the course and an increase in the period between the end and after three months of intervention. Increased long-term subjective well-being was reported and associated with self-care, self-compassion and empathy, as suggested by a number of studies^10^ ^,^ ^44^. The purpose of the practice is not to increase positive affects or reduce negative ones, but to live the present moment, meeting the sensation that arises without creating attachment or aversion and perceiving the experience as it is. A number of studies in socially vulnerable populations show an improvement in emotional regulation and social relationships^23^ ^,^ ^45^. 

The narratives revealed more conscious and more self-compassionate self-observation, as well as insights into changing attitudes and behaviors. The empowerment of being a social activist was noticed, as well as the connection with oneself and with others as a motivation for more people to experience the same situation with greater resilience and without losing focus on self-care, as suggested in a previous study^23^. In addition to that, mind training expands behavioral domains, affecting social behavior outside the training context. According to Freire, this is a process of awareness raising, development of awareness for critical reflection, which includes historical-cultural conditioning and transforms reality^32^ ^-^ ^33^ ^,^ ^53^.

Finally, it is noted that there are significant relationships between the practice of meditation in compassion and situations of social vulnerability, namely: being a black-skinned woman, peripheral and volunteering. In addition, in this research, as cultivating loving reflective awareness, altruistic behavior through connection, perception of emotional self-regulation, clarity of one’s inner values and life purpose. This is explicit in what the literature reports on intersectionality, on the pillars for the promotion of well-being and on the courage to love^11^ ^,^ ^49^. 

These correlations must be interpreted with care due to the specific scenario. It is important to highlight some study limitations regarding the small number of the sample and for being the first study on CBCT® and conducted in a South American cultural context. 

It is worth noting the fact that some participants reported not having performed the practices daily throughout the week. New research studies are needed to deepen the analysis of the effects of this training in longitudinal studies to validate the long-term change in mental and behavioral patterns, in addition to the social impact.

The contribution of this research to the advancement of knowledge and praxis in Nursing and Health can be easily understood, being an innovative study with applicability of a mind training protocol as a (self)care practice related to the singularities of this population’s life, health and disease processes. 

## Conclusion 

The analysis of the effects of applying CBCT® among people in situations of social vulnerability pointed to improved perceptions and management of emotions, feelings, (self)compassion and stress, with impacts on the participants’ individual well-being, peripheral black-skinned women, and the social well-being of their communities, from social activism and engagement to reduce the other’s suffering.

It was also verified that social vulnerability favors low emotional literacy; however, it does not prevent the connection with oneself, in a transforming perspective, from the experience of the meditative practices in compassion, suggesting that CBCT® can constitute an important strategy for the social, emotional and ethical education in the outskirts.
